# Association Between Dietary Betaine Intake and Dyslipidemia in Chinese Children and Adolescents: A Cross-Sectional Study

**DOI:** 10.3390/nu17101742

**Published:** 2025-05-21

**Authors:** Peiliang Chen, Zhitong Xu, Chengping Li, Lianlong Yu, Qianrang Zhu, Zhihao Li, Tao Liu, Dan Liu, Chen Mao

**Affiliations:** 1 Department of Epidemiology, School of Public Health, Southern Medical University, Guangzhou 510515, China; peiliangchen51@hotmail.com (P.C.); zhihaoli2013@smu.edu.cn (Z.L.); 2 Department of Public Health and Preventive Medicine, School of Medicine, Jinan University, Guangzhou 510632, China; winniee@stu2022.jnu.edu.cn (Z.X.); lichengping@stu.jnu.edu.cn (C.L.); gztt_2002@163.com (T.L.); 3 Shandong Center for Disease Control and Prevention, Jinan 250014, China; lianlong00a@163.com; 4 Jiangsu Provincial Center for Disease Control and Prevention, Nanjing 210009, China; zhuqianrang@hotmail.com

**Keywords:** dietary betaine, dyslipidemia, children, adolescent, cross-sectional study

## Abstract

**Background:** Evidence remains limited on the effects of dietary betaine intake and dyslipidemia. We aim to investigate the association between dietary betaine intake and dyslipidemia in Chinese children and adolescents and illustrate the differences in these associations stratified by different food sources. **Methods:** Based on a national cross-sectional study from the China National Nutrition and Health Surveillance of Children and Lactating Mothers, 11,452 individuals aged 6–17 years were enrolled between October 2016 and December 2018. Participants were divided into quartiles according to residual energy-adjusted dietary betaine intake. The associations of dietary betaine with dyslipidemia and lipid profiles were estimated using restricted cubic spline regression and logistic regression analysis. **Results:** Among the 11,452 participants, 2577 (22.5%) individuals were found to have dyslipidemia. The median (IQR) intake of dietary betaine was 56.35 (25.77, 207.66) mg/day. Negative dose-dependent associations were found between residual energy-adjusted dietary betaine intake and dyslipidemia. Compared with participants in the lowest quartile (Q1) of residual energy-adjusted betaine intake, participants in the fourth quartile (Q4) had lower odds of high total cholesterol (TC), high low-density lipoprotein cholesterol (LDL-C), high non-high-density lipoprotein cholesterol (non-HDL-C), high remnant cholesterol (RC), and dyslipidemia, with odds ratios (*OR*) and 95% confidence intervals (95% CI) of 0.56 (0.45, 0.70), 0.65 (0.48, 0.87), 0.53 (0.41, 0.68), 0.42 (0.28, 0.61), and 0.79 (0.69, 0.91), respectively. Furthermore, reduced odds of high TC, high LDL-C, high non-HDL-C, high RC, and dyslipidemia were observed in dietary betaine from plant-source foods but not in animal-source foods. **Conclusions:** High intake of dietary betaine (56.35–207.66 mg/day) was associated with reduced odds of dyslipidemia, including elevated TC, LDL-C, non-HDL-C, and RC, and dietary betaine from plant-source foods revealed significant benefits for dyslipidemia in Chinese children and adolescents.

## 1. Introduction

Dyslipidemia, characterized by elevated total cholesterol (TC), triglycerides (TG), low-density lipoprotein cholesterol (LDL-C), and reduced high-density lipoprotein cholesterol (HDL-C), is a major modifiable risk factor for premature atherosclerosis and early cardiovascular morbidity and mortality [1,2,3]. Startlingly, the prevalence of dyslipidemia among children and adolescents has risen sharply [4], where the prevalence of dyslipidemia among children and adolescents aged 6–17 years was as high as 28.5% in China [5,6], paralleling trends in obesity and metabolic syndrome [7]. Findings have underscored the lifelong dietary strategies targeting lipid regulation in youth to mitigate long-term cardiovascular disease (CVD) risks [8,9].

Betaine, known as trimethylglycine, generated by choline oxidation or delivered through diet, has been reported with a variety of health benefits, such as antioxidant, anti-inflammatory, hepatoprotective, and cardiovascular protective activities [10,11,12,13,14]. Experimental studies in animals demonstrate that betaine supplementation improves lipid metabolism [15,16,17,18]. However, previous studies have focused more on the effects of betaine supplementation on health outcomes by small clinical trials [19,20,21], where betaine interventions may not be similar to the situation of daily dietary intake when comparing the effects. In observational studies, current studies have revealed the associations between dietary betaine intake and cardiovascular or metabolic disease among adults [22,23,24]. Additionally, little human evidence reported associations of dietary betaine intake with dyslipidemia or lipid profiles in children and adolescents, where clarifying the effect of dietary betaine intake on lipid conditions in early life may yield greater cardiovascular benefits. To address this issue, studies on preventing dyslipidemia in pediatric populations with dietary and specific nutrient management warrant further investigation and interpretation in larger population studies.

To further determine whether dietary betaine intake is associated with dyslipidemia among children and adolescents, we conducted a large cross-sectional study from the China National Nutrition and Health Surveillance of Children and Lactating Mothers. Furthermore, we also tried to illustrate the differences in the associations stratified by different food sources.

## 2. Methods

### 2.1. Study Design and Participants

This was a large cross-sectional study from the China National Nutrition and Health Surveillance of Children and Lactating Mothers. The survey was conducted by the National Institute of Nutrition and Health, Chinese Center for Disease Control and Prevention (NINH, China CDC) between October 2016 and December 2018. Five different regions of China were selected for this study, and 15,673 participants were recruited: Northeast, North, Southwest, South, and East, which capture a broad range of dietary patterns, socioeconomic statuses, and lifestyle factors with sufficient variability to reflect the dietary habits of a substantial portion of Chinese children and adolescents. All participants completed a face-to-face interview and physical measurements, and biological samples were collected for biochemical tests. Uniform equipment and methods were used to conduct the surveys. Details of the survey have been described elsewhere [25]. All participants and their guardians had given informed consent in writing to participate in the study. The Ethics Review Board of NINH, China CDC, approved the protocol (No. 201614).

Participants with missing age data or aged beyond 6–17 years (n = 292), those with missing data on blood lipid measures or outcome variables (n = 3695), and those with implausible energy intakes, defined as those in the highest or lowest 1% of the distribution of the ratio of energy intake to estimated energy requirement (EER) [26] based on age and sex (n = 234), were excluded from the analysis. In total, our analysis included 11,452 participants (Figure S1).

### 2.2. Ascertainment of Dietary Data and Betaine Intake

All dietary information for the main analysis was collected by the Food Frequency Questionnaire (FFQ). This standardized questionnaire, developed by experts from NINH, China CDC, has been validated and applied to other large-scale dietary surveys of children in China [27,28,29,30]. Nutrient intake was estimated according to the Standard edition of the *China Food Composition Tables, Standard Edition (6th ed)* [31]. Since there is no information on betaine content in Chinese foods, the dietary intake of total betaine is calculated based on the USDA database [32,33]. To reduce the confounding effects of energy intake, isolate the independent effects of betaine intake, and reduce the impact of multicollinearity, we used the residual method to calculate energy-adjusted betaine intake [34].

### 2.3. Ascertainment of Dyslipidemia

The primary outcome of this study was the prevalence of dyslipidemia. Venous blood samples after 12 h of overnight fasting were collected from participants to measure lipid profiles, namely, total cholesterol (TC), triglycerides (TG), high-density lipoprotein cholesterol (HDL-C), low-density lipoprotein cholesterol (LDL-C), and non-HDL-C. According to the Expert consensus on diagnosis and management of dyslipidemia in children in China [35], elevation in TC (high TC), reduction in HDL-C (low HDL-C), elevation in LDL-C (high LDL-C), and elevation in non-HDL-C (high non-HDL-C) was defined as ≥5.17 mmol/L, <1.03 mmol/L, ≥3.36 mmol/L, and ≥3.74 mmol/L, respectively; elevation in TG (high TG) was defined as ≥1.12 mmol/L, aged 9 years and below, and ≥1.46 mmol/L, aged 10 years and above. Overall, we further defined participants as having “dyslipidemia” if any one of the above definitions were met. On this basis, we calculated remnant cholesterol (RC) as TC minus LDL-C minus HDL-C [36], where elevated remnant cholesterol (high RC) was defined as ≥1.00 mmol/L [37].

### 2.4. Covariates

In this study, we considered various confounding factors that could influence dietary betaine intake: sociodemographic factors (sex, age, nationality, residence, and education level of primary caregivers), lifestyle and dietary habits (physical activity, sleep duration, smoking status, alcohol consumption, total energy, and dietary intake of animal-source foods and plant-source foods), and physical measurement and blood biomarkers (menstruation or spermatorrhea, heart rates, blood pressure, body mass index [BMI], waist circumference, serum total protein, serum albumin, and fasting blood glucose).

Active physical activity was defined as self-reported time spent on activities causing shortness of breath or sweating, averaging more than 1 h per day [38]. Smoking, second-hand smoke exposure, alcohol consumption, and menstruation or spermatorrhea were based on self-reports. BMI was calculated using standardized equipment, with participants categorized as normal weight, overweight, or obese according to criteria specific to sex and age [39]. Red meat, poultry, fishery products, eggs, and dairy products were classified as animal-source foods, while cereals, tuber crops and potatoes, vegetables, fruits, legumes, as well as nuts and peanuts were classified as plant-source foods.

### 2.5. Statistical Analyses

Details of missing covariates are presented in Table S1. We applied multiple imputations by chained equations (MICEs) to address the missing data, creating 10 datasets to leverage their ability to manage both continuous and categorical variables while accommodating complex interrelationships. All variables, including exposure, outcomes, and covariates, were included in the multiple imputation model, supported by our assessment of missing data patterns [40]. The characteristics of participants were presented as the mean (standard deviation [SD]) for continuous variables and the number (percentage [%]) for categorical variables. Participants were divided into quartiles according to residual energy-adjusted dietary betaine intake, and the differences in characteristics of participants across these quartiles were assessed by the Chi-square test, Student’s t-test, or the Wilcoxon rank sum test, as appropriate.

Dose–response associations between betaine intake and dyslipidemia were examined using nonparametrically restricted cubic spline (RCS) regression, with knots positioned at the 25th, 50th, and 75th percentiles to more accurately represent the distribution of dietary betaine. We examined the odds ratios (*OR*s) and 95% confidence intervals (95% CIs) of dyslipidemia according to quartiles of residual energy-adjusted betaine intake using logistic regression analysis. Two sets of models were used. The basic model (model l) was adjusted for sex (male or female), age (continuous), and total energy (continuous). The multivariable model (Model 2) was further adjusted for nationality (Han or others), residence (urban or rural), educational level of the primary caregiver (illiteracy, primary school, junior high school, or high school and above), active physical activity (less than or equal to 60 min per day or greater than 60 min per day), sleep duration (less than 8 h per day, 8–9 h per day, or greater than or equal to 9 h per day), smoking or exposure to second-hand smoke (everyday, 4–6 days per week, 1–3 days per week, less than 1 day per week, or never), alcohol consumption (current, former, or never), menstruation or spermatorrhea (yes or no), intake of animal-source foods (red meat, poultry, fishery products, eggs, and dairy products) (continuous), intake of plant-source foods (cereals, tuber crops and potatoes, vegetables, fruits, legumes, and nuts and peanuts) (continuous), serum albumin (continuous), serum total protein (continuous), fasting blood glucose (continuous), BMI (normal, overweight, or obesity), waist circumference (continuous), systolic blood pressure (continuous), diastolic blood pressure (continuous), and heart rate (continuous). Additionally, the trend test was carried out by taking the median of each residual energy-adjusted betaine intake quartile as a continuous variable in the models.

We further conducted subgroup analyses according to residual energy-adjusted betaine intake from animal or plant-source foods to estimate their association with dyslipidemia separately. We also performed a stratified analysis to examine potential modification effects by different age (6–11 or 12–17 years) and sex (male or female) groups, and the potential modifying effects were assessed by modeling the cross-product term of the stratifying variable with residual energy-adjusted betaine intake, using likelihood ratio tests to estimate interactions.

Finally, to evaluate the robustness of our findings, several sensitivity analyses were performed: (1) we excluded the participants with missing covariates; (2) we further adjusted for more covariates, a history of hypertension (yes or no), diabetes (yes or no), and a family history of hypertension (yes or no) and diabetes (yes or no); (3) we used energy-adjusted dietary betaine intake by calculating as the dietary betaine intake per day divided by the total daily energy intake, to estimate the odds of dyslipidemia; (4) we used residual energy-adjusted betaine by the 24 h dietary recall method over three consecutive days, including two weekdays and one weekend day, to estimate the odds of dyslipidemia. The distribution of dietary betaine data using FFQs and the 24 h dietary recall method is shown in Figure S2.

Analyses were performed using R statistical software (version 4.5.0 for Windows). Statistical tests were two-sided, and *p*-values less than 0.05 were considered statistically significant.

## 3. Results

### 3.1. Characteristics of Participants

As shown in Table 1, of 11,452 participants aged 6–17 years, the mean age was 12.4 years (SD 3.2 years), 64.9% were female, and 90.0% were from the Han ethnic group. The prevalence of overall dyslipidemia among the participants was 22.5%. The average (SD) levels of TG, TC, LDL-C, HDL-C, non-HDL-C, and RC in participants were 4.01 (0.80) mmol/L, 0. 92 (0.60) mmol/L, 2.15 (0.64) mmol/L, 1.46 (0.32) mmol/L, and 2.55 (0.73) mmol/L, and 0.40 (0.31) mmol/L, respectively. In addition to TG, various lipids were different at different levels of betaine intake (*p* < 0.01).

Participants with higher betaine intake were more likely to be older, living in urban areas, and Han nationality, with a higher education level of the primary caregiver (*p* < 0.001); they also had less time for active physical activity, smoked less or were exposed to less second-hand smoke, were more likely to have menstruation or spermatorrhea, and had higher SBP and DBP, higher BMI and WC, higher serum total protein, and higher albumin (*p* < 0.001). Participants with different dietary betaine intakes were significantly different in terms of total energy as well as in their intake of animal-source foods and plant-source foods (*p* < 0.001).

**Figure 1:**
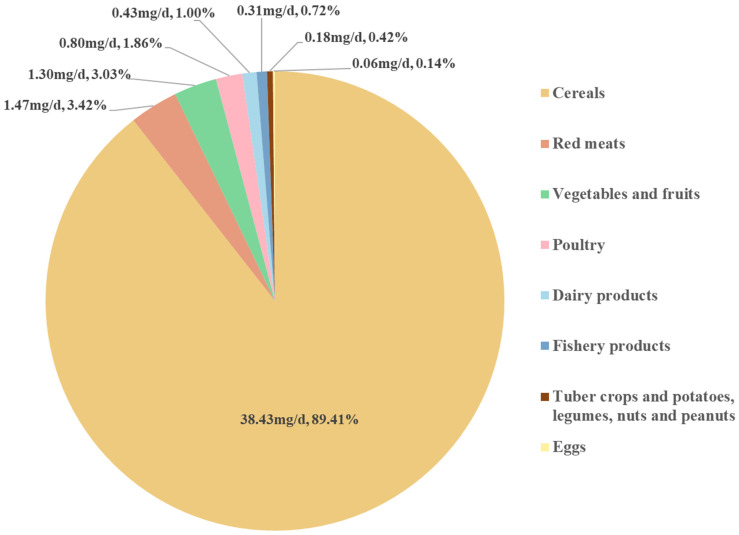
Distribution of dietary betaine intake from different food sources. The numerical values represent the median daily intake of betaine (mg/d) from each food source, and the percentages indicate the proportion of betaine intake contributed by each food source relative to the total intake.

The median (IQR) intake of dietary betaine was 56.35 (25.77, 207.66) mg/day. For the distribution of dietary betaine intake from different sources of foods (Figure 1), cereals contributed the largest proportion (38.43 mg/d, 89.41%), followed by red meat (1.47 mg/d, 3.42%), vegetables and fruits (1.30 mg/d, 3.02%), poultry (0.80 mg/d, 1.86%), and dairy products (0.43 mg/d, 1.00%).

### 3.2. Association Between Dietary Betaine Intake and Dyslipidemia

**Figure 2:**
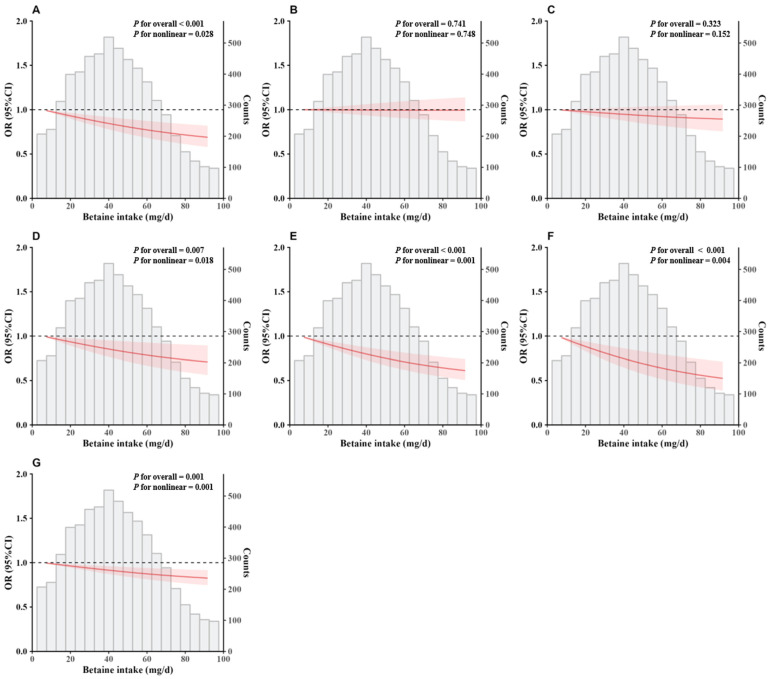
Multiple adjusted and restricted cubic spline models for the associations of residual energy-adjusted dietary betaine intake with high TC (**A**), high TG (**B**), low HDL-C (**C**), high LDL-C (**D**), high non-HDL-C (**E**), high RC (**F**), and dyslipidemia (**G**). The models were adjusted for sex (male or female), age (continuous), total energy (continuous), nationality (Han or others), residence (urban or rural), educational level of the primary caregiver (illiteracy, primary school, junior high school, or high school and above), active physical activity (less than or equal to 60 min per day or greater than 60 min per day), sleep duration (less than 8 h per day, 8–9 h per day, or greater than or equal to 9 h per day), smoking or exposure to second-hand smoke (everyday, 4–6 days per week, 1–3 days per week, less than 1 day per week, or never), alcohol consumption (current, former, or never), menstruation or spermatorrhea (yes or no), intake of animal-source foods (red meat, poultry, fishery products, eggs, and dairy products) (continuous), intake of plant-source foods (cereals, tuber crops and potatoes, vegetables, fruits, legumes, and nuts and peanuts) (continuous), serum albumin (continuous), serum total protein (continuous), fasting blood glucose (continuous), BMI (normal, overweight, or obesity), waist circumference (continuous), systolic blood pressure (continuous), diastolic blood pressure (continuous), and heart rate (continuous).

**Figure 3:**
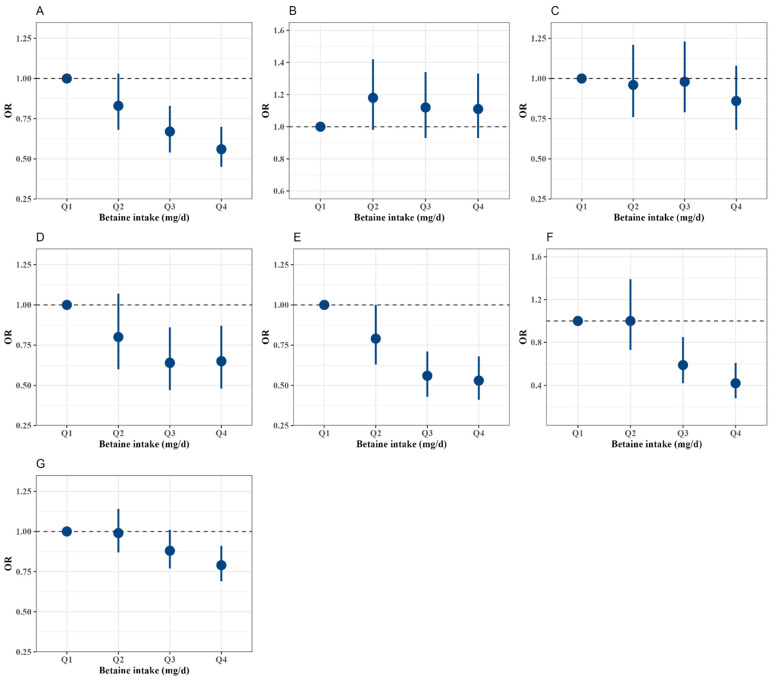
Odds ratios (95% CI) of dyslipidemia at different quartiles of residual energy-adjusted dietary betaine intake in logistic models. High TC (**A**), high TG (**B**), low HDL-C (**C**), high LDL-C (**D**), high non-HDL-C (**E**), high RC (**F**), and dyslipidemia (**G**). The models were adjusted for sex (male or female), age (continuous), and total energy (continuous), nationality (Han or others), residence (urban or rural), educational level of the primary caregiver (illiteracy, primary school, junior high school, or high school and above), active physical activity (less than or equal to 60 min per day or greater than 60 min per day), sleep duration (less than 8 h per day, 8–9 h per day, or greater than or equal to 9 h per day), smoking or exposure to second-hand smoke (everyday, 4–6 days per week, 1–3 days per week, less than 1 day per week, or never), alcohol consumption (current, former, or never), menstruation or spermatorrhea (yes or no), intake of animal-source foods (red meat, poultry, fishery products, eggs, and dairy products) (continuous), intake of plant-source foods (cereals, tuber crops and potatoes, vegetables, fruits, legumes, and nuts and peanuts) (continuous), serum albumin (continuous), serum total protein (continuous), fasting blood glucose (continuous), BMI (normal, overweight, or obesity), waist circumference (continuous), systolic blood pressure (continuous), diastolic blood pressure (continuous), and heart rate (continuous).

Figure 2 and Figure 3 show the dose–response association of residual energy-adjusted betaine intake with dyslipidemia by RCS regression and logistic regression, respectively. In the RCS regression, the residual energy-adjusted betaine intake was negatively associated with high TC, high LDL-C, high non-HDL-C, high RC, and dyslipidemia (*p* < 0.05).

In the multivariable models of logistic regression, compared with participants in the lowest quartile (Q1) of residual energy-adjusted betaine intake, participants in the third to fourth quartiles (Q3 to Q4) had reduced odds of high TC, high LDL-C, high non-HDL-C, high RC, and dyslipidemia (Table 2). The *OR*s (95%*CI*) for high TC, high LDL-C, high non-HDL-C, high RC, and dyslipidemia in Q4 were 0.56 (0.45, 0.70), 0.65 (0.48, 0.87), 0.53 (0.41, 0.68), 0.42 (0.28, 0.61), and 0.79 (0.69, 0.91), respectively. For each quartile increment, the odds for the above-mentioned abnormal lipids decreased by 18.00% (OR, 0.82 [95% CI, 0.77, 0.88]), 14.00% (0.86 [0.78, 0.98]), 26.00% (0.74 [0.65, 0.83]), and 8.00% (0.92 [0.88, 0.96]), with increments per one quartile in residual energy-adjusted betaine intake, respectively (*p* for trend < 0.05).

### 3.3. Subgroup Analyses and Sensitivity Analyses

Table 3 and Table 4 show the stratified results of residual energy-adjusted betaine intake and dyslipidemia by dietary betaine from animal-source foods and plant-source foods after adjustments for sociodemographic factors, lifestyle and dietary habits, physical measurement, and blood biomarkers (results of the basic model are shown in Tables S2 and S3). Similarly, reduced odds of high TC, high LDL-C, high non-HDL-C, high RC, and dyslipidemia were observed in dietary betaine from plant-source foods but not in animal-source foods. To avoid statistical chance, we further analyzed the association between residual energy-adjusted dietary betaine intake from each food and high TC, only to find that betaine intake from poultry, eggs, cereals, and vegetables was negatively associated with high TC (*p* < 0.05), while none had a positive association (Table S4).

Additionally, there was no significant interaction between residual energy-adjusted betaine intake and dyslipidemia (*p* for interaction >0.05), except for RC stratified by sex, where the odds of high RC were lower in females rather than males (*p* for interaction = 0.001) (Table S5).

To verify the stability of the results, several sensitivity analyses were performed. After excluding the participants with missing covariates, adjusting for more covariates, adjusting dietary betaine information by energy density, and using residual energy-adjusted betaine according to the 24 h dietary recall method, no substantial change was observed compared with the results of the main analyses (Tables S6–S9).

## 4. Discussion

In this cross-sectional study of Chinese children and adolescents, we found that a moderate to high increase in betaine intake was positively associated with lower risks of dyslipidemia, in which elevated TC, LDL-C, non-HDL-C, and RC can be used as specific lipid indicators to reflect the above correlation. Dietary betaine from plant-source foods rather than animal-source foods was negatively associated with dyslipidemia in Chinese children and adolescents. These results provided epidemiologic evidence supporting dietary prevention and management of dyslipidemia in children and adolescents, which is of significant public health importance.

Dyslipidemia in children and adolescents has emerged as a growing public health concern. The prevalence of overall dyslipidemia was similar to previous studies [41]. Variations across studies may stem from inconsistent diagnostic thresholds for pediatric dyslipidemia and heterogeneity in dietary exposures, including betaine intake. Although the mechanisms of how dietary betaine affects lipid metabolism remain unclear, the association can be explained in part through animal studies. Animal studies in rodents have demonstrated betaine’s lipid-modulating effects through enhanced hepatic lipid metabolism and reduced oxidative stress [42,43,44], while human evidence remains limited to date. Our study bridges this gap by elucidating the inverse association between dietary betaine intake and dyslipidemia, especially in children and adolescents, mediated partly through lipid-regulating pathways.

Of note, the associations between TC, LDL-C, and non-HDL-C and dietary betaine intake were observed in our study, instead of TG or HDL. This finding was partially supported by previous observational and interventional studies [45,46], but these studies were conducted in adults, and some inverse associations were observed [47]. The differences from the previous results may be due to the characteristics of the study population. In terms of physiological mechanisms, the betaine-homocysteine methyltransferase enzyme would cause increases in the hepatic synthesis of ApoB mRNA and subsequently ApoB synthesis and very-low-density lipoprotein secretion [48,49], which can explain the findings regarding adults that high betaine intake is associated with increases in TC and LDL-C [20,21,47]. However, high dietary betaine intake was negatively associated with elevated TC, LDL-C, and non-HDL-C in children and adolescents, which indicates that the physiological mechanisms may not be fully functioning in pediatric populations compared to the adults, and in-depth studies on the mechanism need further explorations combined with plasma concentrations of betaine. Meanwhile, evidence has pointed out that dyslipidemia served as one of the pediatric risk factors for life-course cardiovascular disease [3], such as elevated LDL-C [50,51,52] and non-HDL-C [53,54], which may infer long-term cardiovascular risk trajectories starting from childhood. From the perspective of preventing CVD, studies have suggested that long-term consumption of betaine may prevent CVD mortality by decreasing inflammation and risk factors [47]. As risk factors for CVD, studies on dyslipidemia and lipid profiles with dietary management may have clinical and public health significance. Our work suggested that betaine in the diet may serve as a modifiable factor for early detection of dyslipidemia, serving as a pilot study for exerting the findings into CVD prevention.

For dietary intake of betaine, previous studies recommended that it should be in the range of 100 to 300 mg daily [55]. The median dietary betaine intake in our study was relatively lower, but it was similar to other studies in the pediatric populations [56], and the odds for dyslipidemia were lower in this range, while the recommended values or adequate intake of betaine for children or adolescents have not been clearly reported yet. There was a large difference in dietary betaine intake due to the different dietary habits of the population in different regions. For food sources of betaine, studies have proved that cereals are the main sources of betaine relative to other foods [57,58,59,60], where China is a large cereal-consuming country with a high dietary intake frequency [61], leading to the largest proportion in our study. Additionally, dietary betaine from plant-source foods was associated with reduced odds of dyslipidemia. Plant-derived betaine may exert superior lipid-lowering effects compared to animal sources due to synergistic interactions with fiber and phytochemicals [62,63]. These mechanisms collectively support our finding that higher betaine intake correlates with favorable lipid profiles, particularly in populations prioritizing plant-based diet patterns.

Therefore, children and adolescents are advised to increase their intake of betaine-rich foods, especially from plant-source foods, which may help improve lipid profiles and reduce the odds of dyslipidemia. We propose that future studies confirm the effects of betaine in large cohorts or interventional studies in this population.

To our knowledge, this is the first large-scale study to evaluate dietary betaine intake and dyslipidemia risk in Chinese children and adolescents. Strengths include a geographically and socioeconomically diverse cohort, rigorous adjustment for energy intake using residual methods, and stratification by betaine sources (plant vs. animal). We employed standardized pediatric cut-offs for TC, LDL-C, non-HDL-C, and RC to enhance clinical relevance. We also controlled for bias by adjusting for lifestyle factors, food intake, and physical measurements. However, limitations warrant consideration. First, capturing varied dietary practices across five provinces, regional agricultural practices, and genetic polymorphisms in betaine metabolism may introduce unmeasured confounding. Second, subjective reporting inaccuracies persist with recall bias in FFQs that were difficult to avoid. We used both FFQs and 3-day 24 h dietary recall data for the analysis of betaine intake and dyslipidemia to minimize the recall bias. Third, although the estimation of dietary betaine intake using the USDA database may not accurately reflect the dietary patterns of the Chinese population, cross-national nutritional assessments have been shown to be feasible based on evidence from previous literature. Additionally, the cross-sectional design precludes causal inference; reverse causality (e.g., dyslipidemia may influence dietary choices) cannot be excluded. Future prospective cohorts should validate these findings, prioritizing direct quantification of betaine metabolites and exploration of gene–diet interactions.

## 5. Conclusions

In conclusion, this large cross-sectional study highlights that high intake of dietary betaine (56.35–207.66 mg/d) was associated with reduced odds of dyslipidemia, namely, high TC, high LDL-C, high non-HDL-C, as well as high RC. The beneficial effect of higher betaine levels on dyslipidemia in this population was more pronounced in the plant-source foods.

## Figures and Tables

**Table 1:** Characteristics of participants grouped by the quartiles of dietary betaine intake.

Characteristics	Overall	Betaine Intake by Quartiles, mg/d Median (IQR): 56.35 (25.77, 207.66)				*p*-Value
		Q1 12.93 (5.53, 20.89)	Q2 39.37 (32.06, 45.20)	Q3 129.26 (77.84, 145.61)	Q4 266.90 (256.20, 386.40)	
**Participants, No.**	11,452	2863	2863	2863	2863	
**Female, No. (%)**	7436 (64.9)	1853 (64.7)	1828(63.8)	1895 (66.2)	1860(65.0)	0.318
**Age, mean (SD), years**	12.4 (3.2)	11.9 (3.2)	12.1 (3.2)	12.4 (3.2)	13.0 (3.1)	<0.001
**Residence, No. (%)**						<0.001
Urban	3880 (37.0)	703 (26.8)	974(37.2)	1068 (40.7)	1135 (43.2)	
Rural	6610 (63.0)	1916 (73.2)	1646 (62.8)	1554 (59.3)	1494 (56.8)	
**Nationality, No. (%)**						<0.001
Han	10,240 (90.0)	2385 (83.7)	2550 (89.6)	2645 (93.1)	2660 (93.8)	
Others	1134 (10.0)	466 (16.3)	295 (10.4)	196 (6.9)	177 (6.2)	
**Educational level of the primary caregiver, No. (%)**						<0.001
Illiteracy	674 (5.9)	234 (8.2)	156 (5.5)	159 (5.6)	125 (4.4)	
Primary school	2928 (25.8)	906 (31.8)	750 (26.4)	641 (22.6)	631 (22.3)	
Junior high school	4228 (37.2)	1044 (36.6)	990 (34.9)	1098 (38.7)	1096 (38.7)	
High school and above	3533 (31.1)	666 (23.4)	943 (33.2)	941 (33.1)	983 (34.7)	
**Active physical activity, No. (%)**						<0.001
≤60 min/d	7993 (76.2)	1857 (73.7)	1966 (75.5)	2059 (76.9)	2111 (78.5)	
>60 min/d	2497 (23.8)	663 (26.3)	637 (24.5)	620 (23.1)	577 (21.5)	
**Sleep duration, No. (%)**						<0.001
<8 h/d	1652 (15.8)	317 (12.6)	378 (14.5)	458 (17.1)	499 (18.6)	
8–9 h/d	2654 (25.3)	571 (22.7)	662 (25.4)	648 (24.2)	773 (28.7)	
≥9 h/d	6182 (58.9)	1632 (64.8)	1564 (60.1)	1569 (58.7)	1417 (52.7)	
**Smoking or exposure to second-hand smoke, No. (%)**						<0.001
Every day	1112 (10.6)	309 (12.3)	303 (11.6)	243 (9.1)	257 (9.6)	
4–6 days/week	409 (3.9)	111 (4.4)	100 (3.8)	108 (4.0)	90 (3.3)	
1–3 days/week	1337 (12.7)	316 (12.5)	356 (13.7)	353 (13.2)	312 (11.6)	
<1 day/week	1648 (15.7)	333 (13.2)	433 (16.6)	436 (16.3)	446 (16.6)	
Never	5988 (57.1)	1451 (57.6)	1412 (54.2)	1539 (57.4)	1586 (58.9)	
**Alcohol consumption, No. (%)**						0.283
Current	477 (4.5)	114 (4.5)	106 (4.1)	116 (4.3)	141 (5.2)	
Former	1100 (10.5)	243 (9.6)	276 (10.6)	295 (11.0)	286 (10.6)	
Never	8918 (85.0)	2163 (85.8)	2222 (85.3)	2269 (84.7)	2264 (84.1)	
**Total energy, mean (SD), kcal/d**	1952.3 (1314.9)	1562.2 (964.6)	1917.3 (1243.5)	2063.3 (1505.6)	2266.6 (1381.9)	<0.001
**Intake of animal-source foods, mean (SD), g/d**						
Red meat	62.4 (68.7)	52.3 (55.8)	64.4 (65.4)	68.3 (78.4)	64.7 (72.1)	<0.001
Poultry	16.1 (36.9)	9.2 (16.0)	17.3 (32.2)	20.5 (49.8)	17.2 (40.2)	<0.001
Fishery products	22.8 (52.0)	13.3 (27.0)	24.1 (42.3)	26.4 (61.7)	27.4 (66.2)	<0.001
Eggs	28.4 (37.1)	23.7 (31.0)	26.3 (33.6)	30.8 (41.0)	32.9 (41.0)	<0.001
Dairy products	137.2 (193.1)	99.3 (131.1)	151.2 (195.9)	156.6 (233.3)	141.7 (192.9)	<0.001
**Intake of plant-source foods, mean (SD), g/d**						
Cereals	12.1 (34.5)	6.4 (21.5)	11.7 (31.5)	15.4 (38.8)	15.0 (41.8)	<0.001
Tuber crops and potatoes	29.8 (60.0)	24.1 (56.1)	33.7 (64.8)	31.1 (57.9)	30.2 (60.4)	<0.001
Vegetable	140.6 (166.7)	112.5 (100.9)	140.3 (152.0)	144.2 (176.6)	165.6 (212.8)	<0.001
Fruits	105.3 (141.2)	88.8 (115.8)	100.0 (133.7)	113.2 (172.9)	119.2 (134.5)	<0.001
Legumes	7.0 (15.2)	5.3 (11.1)	8.2 (17.8)	7.5 (16.2)	7.2 (14.8)	<0.001
Nuts and peanuts	28.3 (72.9)	19.3 (54.8)	30.9 (74.5)	31.7 (80.6)	31.3 (78.0)	<0.001
**Menstruation or spermatorrhea, No. (%)**	4181 (36.5)	895 (31.3)	963 (33.6)	1068 (37.3)	1255 (43.8)	<0.001
**Heart rate, mean (SD), bpm**	88.1 (13.4)	88.5 (13.2)	88.0 (13.2)	88.1 (13.9)	87.9 (13.2)	0.448
**Systolic blood pressure, mean (SD), mmHg**	112.1 (11.9)	110.6 (11.3)	110.8 (11.2)	112.3 (12.0)	114.9 (12.4)	<0.001
**Diastolic blood pressure, mean (SD), mmHg**	66.3 (8.6)	65.4 (8.5)	65.4 (8.4)	66.3 (8.3)	68.0 (8.9)	<0.001
**BMI, mean (SD), kg/m^2^**	18.7 (4.6)	18.1 (4.1)	18.4 (4.3)	18.8 (4.4)	19.5 (5.4)	<0.001
Normal, No. (%)	9374 (81.9)	2419 (84.6)	2367 (82.7)	2335 (81.6)	2253 (78.7)	
Overweight, No. (%)	1179 (10.3)	260 (9.1)	295 (10.3)	295 (10.3)	329 (11.5)	
Obesity, No. (%)	894 (7.8)	181 (6.3)	201 (7.0)	232 (8.1)	280 (9.8)	
**Waist circumference, mean (SD), cm**	63.7 (11.1)	61.9 (10.9)	63.0 (10.8)	64.0 (11.1)	65.9 (11.4)	<0.001
**Serum biomarkers, mean (SD)**						
Total protein, g/L	76.2 (5.1)	75.9 (5.0)	76.0 (5.1)	76.0 (5.2)	76.7 (5.1)	<0.001
Albumin, g/L	49.3 (3.0)	49.1 (3.0)	49.1 (3.0)	49.3 (3.0)	49.6 (3.0)	<0.001
Fasting blood glucose, mmol/L	5.15 (0.58)	5.13 (0.62)	5.17 (0.61)	5.15 (0.54)	5.17 (0.56)	0.016
TC, mmol/L	4.01 (0.80)	4.04 (0.80)	4.05 (0.86)	4.01 (0.77)	3.96 (0.76)	<0.001
TG, mmol/L	0.92 (0.60)	0.92 (0.42)	0.91 (0.39)	0.93 (0.96)	0.94 (0.41)	0.299
HDL-C, mmol/L	1.46 (0.32)	1.45 (0.31)	1.46 (0.32)	1.48 (0.32)	1.48 (0.33)	0.001
LDL-C, mmol/L	2.15 (0.64)	2.17 (0.62)	2.17 (0.67)	2.13 (0.63)	2.12 (0.63)	0.007
Non-HDL-C, mmol/L	2.55 (0.73)	2.59 (0.73)	2.59 (0.79)	2.53 (0.70)	2.49 (0.70)	<0.001
RC, mmol/L	0.40 (0.31)	0.42 (0.30)	0.42 (0.37)	0.40 (0.28)	0.36 (0.26)	<0.001
**Dyslipidemia, No. (%)**	2577 (22.5)	683 (23.9)	669 (23.4)	631 (22.0)	594 (20.7)	0.022

Continuous variables are shown as mean (standard deviation); categorical variables are shown as the number of cases (percentage). Betaine intake and its quartiles are presented as median (interquartile range). TC: total cholesterol; TG: triglyceride; HDL-C: high-density lipoprotein cholesterol; LDL-C: low-density lipoprotein cholesterol; non-HDL-C: non-high-density lipoprotein cholesterol; RC: remnant cholesterol.

**Table 2:** Association between residual energy-adjusted dietary betaine intake and dyslipidemia.

Variables	Residual Energy-Adjusted Betaine Intake by Quartiles *				Increments per 1 Quartile *	*p*_trend_
	Q1	Q2 *	Q3 *	Q4 *		
High TC						
Cases/N	239/2863	251/2863	198/2863	162/2863		
Model 1	1.00 (Ref.)	0.97 (0.80, 1.19)	0.79 (0.64, 0.97)	0.69 (0.56, 0.86)	0.88 (0.82, 0.94)	<0.001
Model 2	1.00 (Ref.)	0.83 (0.68, 1.03)	0.67 (0.54, 0.83)	0.56 (0.45, 0.70)	0.82 (0.77, 0.88)	<0.001
High TG						
Cases/N	278/2863	358/2863	349/2863	347/2863		
Model 1	1.00 (Ref.)	1.21 (1.01, 1.44)	1.21 (1.02, 1.44)	1.28 (1.08, 1.52)	1.07 (1.02, 1.13)	0.008
Model 2	1.00 (Ref.)	1.18 (0.98, 1.42)	1.12 (0.93, 1.34)	1.11 (0.93, 1.33)	1.02 (0.97, 1.08)	0.459
Low HDL-C						
Cases/N	212/2863	164/2863	178/2863	159/2863		
Model 1	1.00 (Ref.)	0.86 (0.69, 1.08)	0.90 (0.73, 1.12)	0.75 (0.60, 0.93)	0.92 (0.86, 0.99)	0.018
Model 2	1.00 (Ref.)	0.96 (0.76, 1.21)	0.98 (0.79, 1.23)	0.86 (0.68, 1.08)	0.96 (0.89, 1.03)	0.255
High LDL-C						
Cases/N	117/2863	117/2863	95/2863	98/2863		
Model 1	1.00 (Ref.)	0.92 (0.69, 1.22)	0.77 (0.58, 1.03)	0.86 (0.65, 1.13)	0.92 (0.86, 0.99)	0.018
Model 2	1.00 (Ref.)	0.80 (0.60, 1.07)	0.64 (0.47, 0.86)	0.65 (0.48, 0.87)	0.86 (0.78, 0.94)	0.001
High non-HDL-C						
Cases/N	196/2863	187/2863	133/2863	128/2863		
Model 1	1.00 (Ref.)	0.91 (0.73, 1.14)	0.66 (0.52, 0.83)	0.67 (0.53, 0.85)	0.86 (0.79, 0.92)	<0.001
Model 2	1.00 (Ref.)	0.79 (0.63, 1.00)	0.56 (0.43, 0.71)	0.53 (0.41, 0.68)	0.79 (0.73, 0.86)	<0.001
High RC						
Cases/N	88/2863	99/2863	59/2863	40/2863		
Model 1	1.00 (Ref.)	1.06 (0.77, 1.45)	0.64 (0.45, 0.90)	0.45 (0.30, 0.65)	0.76 (0.68, 0.84)	<0.001
Model 2	1.00 (Ref.)	1.00 (0.73, 1.39)	0.59 (0.42, 0.85)	0.42 (0.28, 0.61)	0.74 (0.65, 0.83)	<0.001
Dyslipidemia						
Cases/N	664/2863	693/2863	636/2863	584/2863		
Model 1	1.00 (Ref.)	1.02 (0.89, 1.16)	0.93 (0.82, 1.06)	0.88 (0.77, 1.00)	0.95 (0.92, 0.99)	0.018
Model 2	1.00 (Ref.)	0.99 (0.87, 1.14)	0.88 (0.77, 1.01)	0.79 (0.69, 0.91)	0.92 (0.88, 0.96)	<0.001

* Estimated as odds ratios (ORs) and 95% confidence intervals (95% CIs). TC: total cholesterol; TG: triglyceride; HDL-C: high-density lipoprotein cholesterol; LDL-C: low-density lipoprotein cholesterol; non-HDL-C: non-high-density lipoprotein cholesterol; RC: remnant cholesterol. Model 1 was adjusted for sex (male or female), age (continuous), and total energy (continuous). Model 2 was further adjusted for nationality (Han or others), residence (urban or rural), educational level of the primary caregiver (illiteracy, primary school, junior high school, or high school and above), active physical activity (less than or equal to 60 min per day or greater than 60 min per day), sleep duration (less than 8 h per day, 8–9 h per day, or greater than or equal to 9 h per day), smoking or exposure to second-hand smoke (everyday, 4–6 days per week, 1–3 days per week, less than 1 day per week, or never), alcohol consumption (current, former, or never), menstruation or spermatorrhea (yes or no), intake of animal-source foods (red meat, poultry, fishery products, eggs, and dairy products) (continuous), intake of plant-source foods (cereals, tuber crops and potatoes, vegetables, fruits, legumes, and nuts and peanuts) (continuous), serum albumin (continuous), serum total protein (continuous), fasting blood glucose (continuous), BMI (normal, overweight, or obesity), waist circumference (continuous), systolic blood pressure (continuous), diastolic blood pressure (continuous), and heart rate (continuous).

**Table 3:** Association between residual energy-adjusted dietary betaine intake from animal-source foods and dyslipidemia.

Variables	Residual Energy-Adjusted Betaine Intake from Animal-Source Foods by Quartiles				Increments per 1 Quartile	*p*_trend_
	Q1	Q2	Q3	Q4		
High TC						
Cases/N	167/2863	213/2863	238/2863	232/2863		
OR (95% CIs)	1.00 (Ref.)	1.17 (0.94, 1.46)	1.23 (0.99, 1.53)	1.16 (0.92, 1.46)	1.05 (0.98, 1.13)	0.187
High TG						
Cases/N	336/2863	352/2863	309/2863	335/2863		
OR (95% CIs)	1.00 (Ref.)	0.96 (0.81, 1.14)	0.85 (0.71, 1.01)	0.98 (0.81, 1.19)	0.98 (0.92, 1.04)	0.484
Low HDL-C						
Cases/N	208/2863	184/2863	168/2863	153/2863		
OR (95% CIs)	1.00 (Ref.)	0.91 (0.73, 1.13)	0.80 (0.64, 1.01)	0.72 (0.56, 0.93)	0.90 (0.83, 0.97)	0.007
High LDL-C						
Cases/N	86/2863	106/2863	127/2863	108/2863		
OR (95% CIs)	1.00 (Ref.)	1.13 (0.83, 1.53)	1.31 (0.98, 1.76)	1.09 (0.79, 1.51)	1.04 (0.94, 1.15)	0.398
High non-HDL-C						
Cases/N	139/2863	158/2863	180/2863	167/2863		
OR (95% CIs)	1.00 (Ref.)	0.98 (0.77, 1.26)	1.07 (0.84, 1.37)	0.98 (0.75, 1.27)	1.00 (0.92, 1.09)	0.925
High RC						
Cases/N	70/2863	80/2863	76/2863	60/2863		
OR (95% CIs)	1.00 (Ref.)	1.05 (0.75, 1.48)	0.99 (0.70, 1.40)	0.69 (0.46, 1.02)	0.90 (0.80, 1.01)	0.083
Dyslipidemia						
Cases/N	628/2863	662/2863	643/2863	644/2863		
OR (95% CIs)	1.00 (Ref.)	1.01 (0.89, 1.16)	0.97 (0.85, 1.11)	0.97 (0.84, 1.12)	0.99 (0.94, 1.03)	0.552

TC: total cholesterol; TG: triglyceride; HDL-C: high-density lipoprotein cholesterol; LDL-C: low-density lipoprotein cholesterol; non-HDL-C: non-high-density lipoprotein cholesterol; RC: remnant cholesterol; OR: odds ratio; CI: confidence interval. The models were adjusted for sex (male or female), age (continuous), total energy (continuous), nationality (Han or others), residence (urban or rural), educational level of the primary caregiver (illiteracy, primary school, junior high school, or high school and above), active physical activity (less than or equal to 60 min per day or greater than 60 min per day), sleep duration (less than 8 h per day, 8–9 h per day, or greater than or equal to 9 h per day), smoking or exposure to second-hand smoke (everyday, 4–6 days per week, 1–3 days per week, less than 1 day per week, or never), alcohol consumption (current, former, or never), menstruation or spermatorrhea (yes or no), intake of animal-source foods (red meat, poultry, fishery products, eggs, and dairy products) (continuous), intake of plant-source foods (cereals, tuber crops and potatoes, vegetables, fruits, legumes, and nuts and peanuts) (continuous), serum albumin (continuous), serum total protein (continuous), fasting blood glucose (continuous), BMI (normal, overweight, or obesity), waist circumference (continuous), systolic blood pressure (continuous), diastolic blood pressure (continuous), and heart rate (continuous).

**Table 4:** Association between residual energy-adjusted dietary betaine intake from plant-source foods and dyslipidemia.

Variables	Residual Energy-Adjusted Betaine Intake from Plant-Source Foods by Quartiles				Increments per 1 Quartile	*p*_trend_
	Q1	Q2	Q3	Q4		
High TC						
Cases/N	230/2863	281/2863	179/2863	160/2863		
OR (95% CIs)	1.00 (Ref.)	1.15 (0.94, 1.41)	0.70 (0.56, 0.86)	0.65 (0.52, 0.82)	0.84 (0.78, 0.90)	<0.001
High TG						
Cases/N	313/2863	336/2863	340/2863	343/2863		
OR (95% CIs)	1.00 (Ref.)	0.96 (0.81, 1.15)	0.94 (0.79, 1.12)	0.99 (0.83, 1.18)	0.99 (0.94, 1.05)	0.849
Low HDL-C						
Cases/N	184/2863	191/2863	178/2863	160/2863		
OR (95% CIs)	1.00 (Ref.)	1.23 (0.97, 1.54)	1.16 (0.92, 1.46)	0.99 (0.78, 1.25)	0.99 (0.92, 1.06)	0.775
High LDL-C						
Cases/N	121/2863	123/2863	89/2863	94/2863		
OR (95% CIs)	1.00 (Ref.)	0.91 (0.69, 1.19)	0.62 (0.46, 0.83)	0.66 (0.49, 0.89)	0.85 (0.77, 0.93)	<0.001
High non-HDL-C						
Cases/N	184/2863	212/2863	123/2863	125/2863		
OR (95% CIs)	1.00 (Ref.)	1.10 (0.88, 1.37)	0.60 (0.47, 0.7)	0.63 (0.49, 0.80)	0.82 (0.75, 0.88)	<0.001
High RC						
Cases/N	86/2863	101/2863	55/2863	44/2863		
OR (95% CIs)	1.00 (Ref.)	1.12 (0.82, 1.53)	0.59 (0.41, 0.85)	0.50 (0.34, 0.73)	0.77 (0.68, 0.86)	<0.001
Dyslipidemia						
Cases/N	648/2863	727/2863	617/2863	585/2863		
OR (95% CIs)	1.00 (Ref.)	1.12 (0.98, 1.28)	0.90 (0.78, 1.02)	0.86 (0.75, 0.98)	0.93 (0.89, 0.97)	0.001

TC: total cholesterol; TG: triglyceride; HDL-C: high-density lipoprotein cholesterol; LDL-C: low-density lipoprotein cholesterol; non-HDL-C: non-high-density lipoprotein cholesterol; RC: remnant cholesterol; OR: odds ratio; CI: confidence interval. The models were adjusted for sex (male or female), age (continuous), total energy (continuous), nationality (Han or others), residence (urban or rural), educational level of the primary caregiver (illiteracy, primary school, junior high school, or high school and above), active physical activity (less than or equal to 60 min per day or greater than 60 min per day), sleep duration (less than 8 h per day, 8–9 h per day, or greater than or equal to 9 h per day), smoking or exposure to second-hand smoke (everyday, 4–6 days per week, 1–3 days per week, less than 1 day per week, or never), alcohol consumption (current, former, or never), menstruation or spermatorrhea (yes or no), intake of animal-source foods (red meat, poultry, fishery products, eggs, and dairy products) (continuous), intake of plant-source foods (cereals, tuber crops and potatoes, vegetables, fruits, legumes, and nuts and peanuts) (continuous), serum albumin (continuous), serum total protein (continuous), fasting blood glucose (continuous), BMI (normal, overweight, or obesity), waist circumference (continuous), systolic blood pressure (continuous), diastolic blood pressure (continuous), and heart rate (continuous).

## Data Availability

There are restrictions on the availability of data for the study. In accordance with the policy of the National Institute for Nutrition and Health, Chinese Center for Disease Control and Prevention, the data related to this research cannot be publicly disclosed. However, interested parties can contact the corresponding authors to request access to the data or source code of this study.

## Supplementary Materials

The following supporting information can be downloaded at: https://www.mdpi.com/article/10.3390/nu17101742/s1, Figure S1. Flowchart of data sources and reasons for exclusions; Figure S2. Distribution of dietary betaine data using FFQs and 24-h dietary recalls method; Table S1. The numbers of participants with missing covariates; Table S2. Association between residual energy-adjusted dietary betaine intake from animal source foods and dyslipidemia; Table S3. Association between residual energy-adjusted dietary betaine intake from plant source foods and dyslipidemia; Table S4. The association between residual energy-adjusted dietary betaine intake from each food and High TC; Table S5. The associations of residual energy-adjusted dietary betaine intake with dyslipidemia stratified by age groups and sex; Table S6. Odds ratios (95% CIs) of residual energy-adjusted dietary betaine intake with dyslipidemia after excluding the participants with missing data of covariates: sensitivity analyses; Table S7. Odds ratios (95% CIs) of residual energy-adjusted dietary betaine intake with dyslipidemia after adjusting more covariates: sensitivity analyses; Table S8. Odds ratios (95% CIs) of energy-adjusted dietary betaine intake with dyslipidemia: sensitivity analyses; Table S9. Odds ratios (95% CIs) of residual energy-adjusted dietary betaine intake with dyslipidemia using 24-h dietary recalls method: sensitivity analyses.

## Abbreviations

TG: triglyceride; TC: total cholesterol; HDL-C: high-density lipoprotein cholesterol; LDL-C: low-density lipoprotein cholesterol; non-HDL-C: non-high-density lipoprotein cholesterol; RC: remnant cholesterol; CVD: cardiovascular disease; NINH, China CDC: National Institute of Nutrition and Health, Chinese Center for Disease Control and Prevention; EER: estimated energy requirement; FFQ: Food Frequency Questionnaire; BMI: body mass index; MICE: multiple imputation by chained equations; SD: standard deviation; RCS: restricted cubic spline; OR: odds ratio; CI: confidence interval.
