# Supplementary material for: Association Between Dietary Betaine Intake and Dyslipidemia in Chinese Children and Adolescents: A Cross-Sectional Study
Source: Nutrients. 2025 May 21;17(10):1742. doi: 10.3390/nu17101742 (PMC12113887; doi:10.3390/nu17101742)
Supplement: Supplementary file 1 [file nutrients-17-01742-s001.zip › nutrients-3626777-supplementary.pdf]

Contents

Figure S1. Flowchart of data sources and reasons for exclusions 1

Figure S2. Distribution of dietary betaine data using FFQs and 24-hour dietary recalls method .. 2

Table S1 The numbers of participants with missing covariates 3

Table S2 Association between residual energy-adjusted dietary betaine intake from animal source foods and dyslipidemia 4

Table S3 Association between residual energy-adjusted dietary betaine intake from plant source foods and dyslipidemia 5

Table S4 The association between residual energy-adjusted dietary betaine intake from each food and High TC 6

Table S5 The associations of residual energy-adjusted dietary betaine intake with dyslipidemia stratified by age groups and sex 7

Table S6 Odds ratios (95% CIs) of residual energy-adjusted dietary betaine intake with dyslipidemia after excluding the participants with missing data of covariates: sensitivity analyses 9

Table S7 Odds ratios (95% CIs) of residual energy-adjusted dietary betaine intake with dyslipidemia after adjusting more covariates: sensitivity analyses 10

Table S8 Odds ratios (95% CIs) of energy-adjusted dietary betaine intake with dyslipidemia: sensitivity analyses 11

Table S9 Odds ratios (95% CIs) of residual energy-adjusted dietary betaine intake with dyslipidemia using 24-h dietary recalls method: sensitivity analyses 12

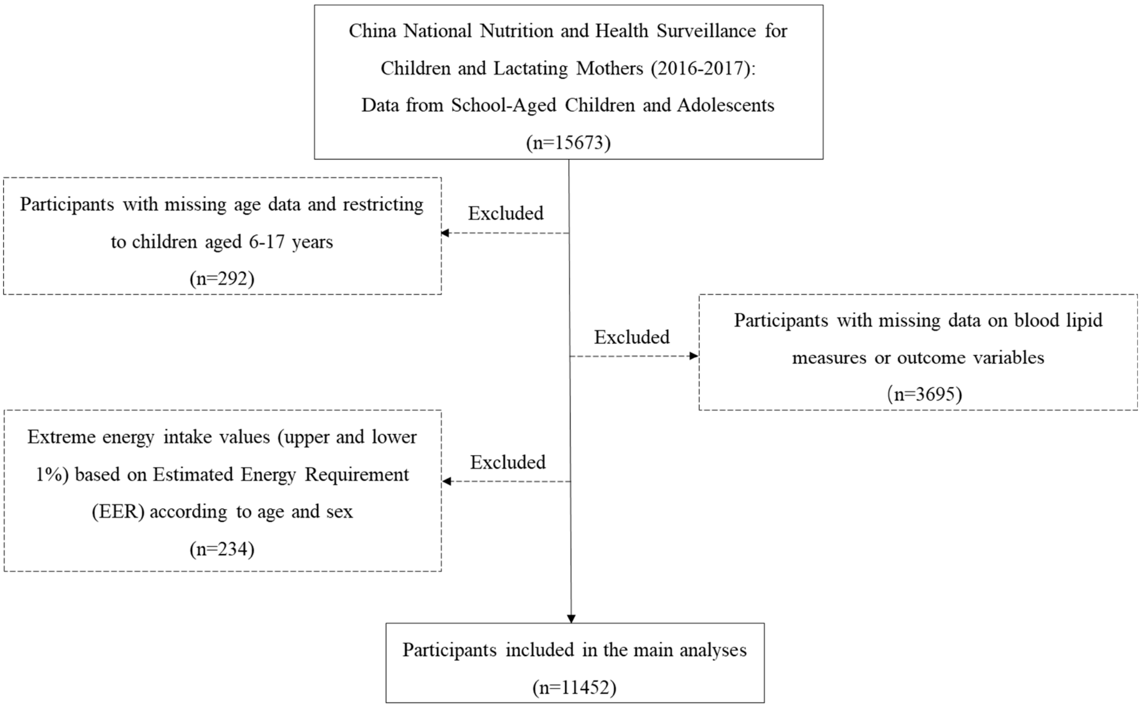

Figure S1. Flowchart of data sources and reasons for exclusions

Since it was inevitable that some extreme values may occur due to recall and measurement bias. we excluded extreme values that may distort the statistical analysis results, to more accurately reflect the energy intake distribution of the population.

In consideration that children and adolescents are in a stage of vigorous growth and development, their energy requirements gradually increase with age, and disparities with certain gender. The calculation formula of estimated energy requirement (EER) was performed in the inclusion of participants, taking into account several factors to predict the average daily dietary energy intake required to maintain energy balance, such as age-dependent physical activity levels, growth needs, and physiological stages. By comparing the actual intake with EER, the nutritional status of individuals can be evaluated to determine if it is appropriate. Participants with implausible energy intakes defined as those in the highest or lowest 1% of the distribution of the ratio of energy intake to EER.

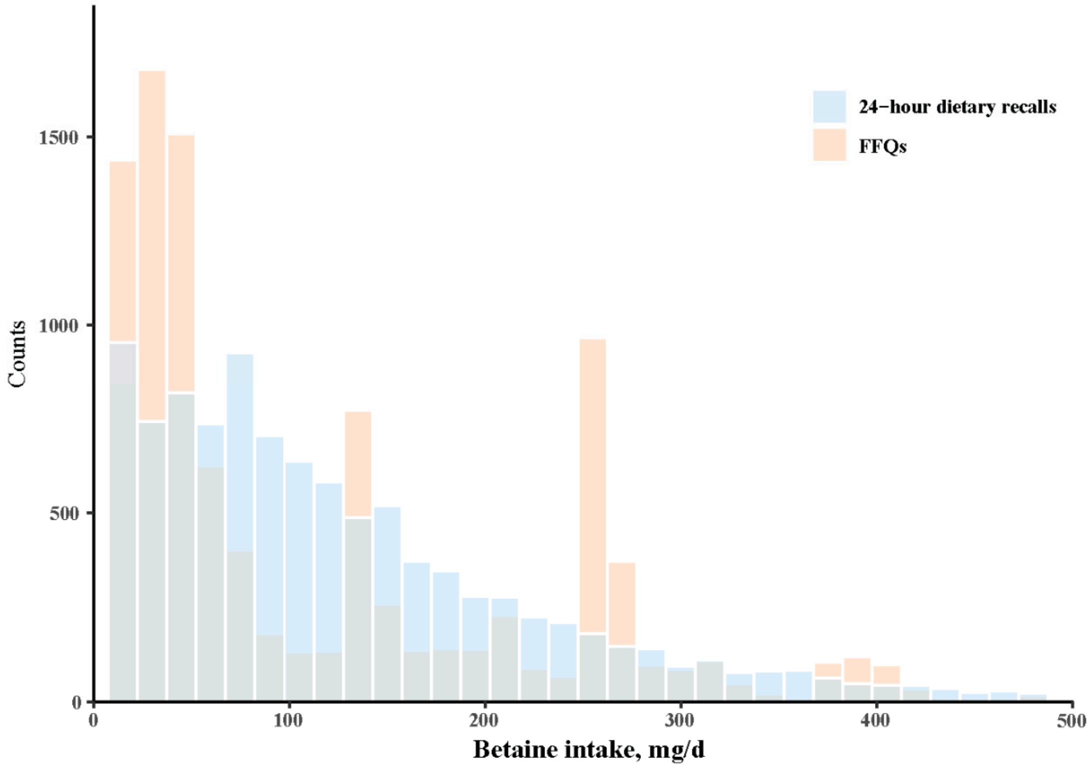

Figure S2. Distribution of dietary betaine data using FFQs and 24-hour dietary recalls method

Table S1 The numbers of participants with missing covariates

Covariates	n	Percentage (%)
Residence	962	8.40
Nationality	78	0.68
Educational level of the primary caregiver	89	0.78
Active physical activity	962	8.40
Sleep duration	964	8.42
Smoking or exposure to second-hand smoke	958	8.37
Alcohol consumption	957	8.36
Fruit	2	0.02
Poultry	1	0.01
Heart rates	12	0.10
Systolic blood pressure	8	0.07
Diastolic blood pressure	8	0.07
BMI	5	0.04
Waist circumference	21	0.18
Albumin	1	0.01

Table S2 Association between residual energy-adjusted dietary betaine intake from animal source foods and dyslipidemia

Variables	Residual energy-adjusted betaine intake from animal source foods by quartiles				Increments per 1 quartile*	P_{trend}
	Q1	Q2	Q3	Q4		
**High TC**						
Cases/N	167/2863	213/2863	238/2863	232/2863		
OR (95% CIs)	1.00 (Ref.)	1.28 (1.03, 1.58)	1.49 (1.21, 1.83)	1.50 (1.22, 1.85)	1.14 (1.07, 1.22)	<0.001
**High TG**						
Cases/N	336/2863	352/2863	309/2863	335/2863		
OR (95% CIs)	1.00 (Ref.)	1.03 (0.87, 1.21)	0.92 (0.78, 1.09)	1.05 (0.89, 1.23)	1.00 (0.95, 1.06)	0.912
**Low HDL-C**						
Cases/N	208/2863	184/2863	168/2863	153/2863		
OR (95% CIs)	1.00 (Ref.)	0.91 (0.74, 1.12)	0.79 (0.64, 0.97)	0.68 (0.55, 0.84)	0.88 (0.82, 0.94)	<0.001
**High LDL-C**						
Cases/N	86/2863	106/2863	127/2863	108/2863		
OR (95% CIs)	1.00 (Ref.)	1.22 (0.91, 1.64)	1.52 (1.15, 2.01)	1.32 (0.99, 1.76)	1.11 (1.01, 1.21)	0.024
**High non-HDL-C**						
Cases/N	139/2863	158/2863	180/2863	167/2863		
OR (95% CIs)	1.00 (Ref.)	1.14 (0.90, 1.44)	1.33 (1.06, 1.67)	1.25 (0.99, 1.58)	1.08 (1.01, 1.17)	0.028
**High RC**						
Cases/N	70/2863	80/2863	76/2863	60/2863		
OR (95% CIs)	1.00 (Ref.)	1.13 (0.81, 1.56)	1.09 (0.78, 1.51)	0.87 (0.61, 1.24)	0.96 (0.86, 1.07)	0.456
**Dyslipidemia**						
Cases/N	628/2863	662/2863	643/2863	644/2863		
OR (95% CIs)	1.00 (Ref.)	1.06 (0.94, 1.20)	1.04 (0.92, 1.18)	1.06 (0.93, 1.20)	1.02 (0.98, 1.06)	0.448

TC: total cholesterol; TG: triglyceride; HDL-C: high-density lipoprotein cholesterol; LDL-C: low-density lipoprotein cholesterol; non-HDL-C: non-high-density lipoprotein cholesterol; RC: remnant cholesterol; OR: odds ratio; CI: confidence interval.

The models were adjusted for sex (male or female), age (continuous), and total energy (continuous).

Table S3 Association between residual energy-adjusted dietary betaine intake from plant source foods and dyslipidemia

Variables	Residual energy-adjusted betaine intake from plant source foods by quartiles				Increments per 1 quartile*	P_{trend}
	Q1	Q2	Q3	Q4		
**High TC**						
Cases/N	230/2863	281/2863	179/2863	160/2863		
OR (95% CIs)	1.00 (Ref.)	1.15 (0.96, 1.39)	0.74 (0.60, 0.91)	0.71 (0.57, 0.87)	0.86 (0.81, 0.92)	<0.001
**High TG**						
Cases/N	313/2863	336/2863	340/2863	343/2863		
OR (95% CIs)	1.00 (Ref.)	0.98 (0.83, 1.16)	1.02 (0.86, 1.20)	1.11 (0.94, 1.30)	1.04 (0.98, 1.09)	0.186
**Low HDL-C**						
Cases/N	184/2863	191/2863	178/2863	160/2863		
OR (95% CIs)	1.00 (Ref.)	1.16 (0.94, 1.44)	1.05 (0.85, 1.31)	0.87 (0.70, 1.08)	0.95 (0.89, 1.02)	0.147
**High LDL-C**						
Cases/N	121/2863	123/2863	89/2863	94/2863		
OR (95% CIs)	1.00 (Ref.)	0.94 (0.72, 1.23)	0.70 (0.53, 0.93)	0.79 (0.60, 1.04)	0.90 (0.83, 0.99)	0.024
**High non-HDL-C**						
Cases/N	184/2863	212/2863	123/2863	125/2863		
OR (95% CIs)	1.00 (Ref.)	1.11 (0.90, 1.38)	0.65 (0.51, 0.82)	0.69 (0.55, 0.88)	0.85 (0.79, 0.91)	<0.001
**High RC**						
Cases/N	86/2863	101/2863	55/2863	44/2863		
OR (95% CIs)	1.00 (Ref.)	1.12 (0.83, 1.52)	0.62 (0.43, 0.87)	0.51 (0.35, 0.73)	0.78 (0.70, 0.87)	<0.001
**Dyslipidemia**						
Cases/N	648/2863	727/2863	617/2863	585/2863		
OR (95% CIs)	1.00 (Ref.)	1.12 (0.99, 1.27)	0.92 (0.81, 1.05)	0.90 (0.79, 1.02)	0.95 (0.91, 0.99)	0.013

TC: total cholesterol; TG: triglyceride; HDL-C: high-density lipoprotein cholesterol; LDL-C: low-density lipoprotein cholesterol; non-HDL-C: non-high-density lipoprotein cholesterol; RC: remnant cholesterol; OR: odds ratio; CI: confidence interval.

The models were adjusted for sex (male or female), age (continuous), and total energy (continuous).

Table S4 The association between residual energy-adjusted dietary betaine intake from each food and High TC

Variables	Residual energy-adjusted betaine intake by quartiles				Increments per 1 quartile	P_{trend}
	Q1	Q2	Q3	Q4		
**Animal source foods**						
Red meat, cases/N	217/2863	201/2803	217/2914	215/2872		
OR (95% CIs)	1.00 (Ref.)	0.91 (0.73, 1.13)	0.90 (0.72, 1.13)	0.92 (0.74, 1.13)	0.97 (0.91, 1.04)	0.450
Poultry, cases/N	221/2608	227/2959	242/2793	160/3901		
OR (95% CIs)	1.00 (Ref.)	0.82 (0.66, 1.03)	0.96 (0.76, 1.20)	0.68 (0.53, 0.87)	0.91 (0.84, 0.98)	0.019
Fishery products, cases/N	218/2855	230/2871	14/181	388/5545		
OR (95% CIs)	1.00 (Ref.)	1.06 (0.86, 1.30)	1.06 (0.57, 1.83)	1.04 (0.87, 1.25)	1.01 (0.95, 1.07)	0.785
Dairy products, cases/N	190/2859	195/2855	229/2769	236/2969		
OR (95% CIs)	1.00 (Ref.)	1.02 (0.82, 1.26)	1.10 (0.89, 1.37)	1.16 (0.90, 1.50)	1.05 (0.97, 1.14)	0.216
Eggs, cases/N	256/2863	188/2403	202/3077	204/3109		
OR (95% CIs)	1.00 (Ref.)	0.82 (0.66, 1.00)	0.72 (0.59, 0.88)	0.67 (0.54, 0.85)	0.87 (0.81, 0.93)	<0.001
**Plant source foods**						
Cereals, cases/N	193/2845	303/2878	195/2826	159/2903		
OR (95% CIs)	1.00 (Ref.)	1.40 (1.15, 1.71)	0.88 (0.71, 1.09)	0.72 (0.57, 0.90)	0.87 (0.81, 0.93)	<0.001
Tuber crops and potatoes, cases/N	72/927	376/4660	197/3000	205/2865		
OR (95% CIs)	1.00 (Ref.)	1.06 (0.80, 1.43)	0.83 (0.62, 1.13)	0.99 (0.75, 1.34)	0.96 (0.88, 1.04)	0.286
Vegetables, cases/N	228/2860	150/1676	281/4053	191/2863		
OR (95% CIs)	1.00 (Ref.)	1.15 (0.92, 1.43)	0.84 (0.70, 1.02)	0.82 (0.65, 1.03)	0.92 (0.85, 0.99)	0.020
Fruits, cases/N	222/2853	204/2811	192/2925	232/2863		
OR (95% CIs)	1.00 (Ref.)	1.02 (0.82, 1.27)	0.98 (0.77, 1.23)	1.16 (0.93, 1.44)	1.04 (0.97, 1.12)	0.222
Legumes, cases/N	191/2340	214/3208	202/2922	243/2982		
OR (95% CIs)	1.00 (Ref.)	0.98 (0.79, 1.21)	0.91 (0.74, 1.13)	1.02 (0.83, 1.25)	1.00 (0.94, 1.07)	0.997
Nuts and peanuts, cases/N	230/2694	-	-	620/8758		
OR (95% CIs)	1.00 (Ref.)	-	-	0.93 (0.79, 1.10)	0.98 (0.92, 1.03)	0.400

The models were adjusted for sex (male or female), age (continuous), total energy (continuous), nationality (Han or others), residence (urban or rural), educational level of the primary caregiver (illiteracy, primary school, junior high school or high school, and above), active physical activity (less than or equal to 60 minutes per day, greater than 60 minutes per day), sleep duration (less than 8 hours per day, 8-9 hours per day, greater than or equal to 9 hours per day), smoking or exposure to second-hand smoke (everyday, 4–6 days per week, 1–3 days per week, less than 1 day per week or never), alcohol consumption (current, former, never), menstruation or spermatorrhea (yes or no), intake of animal source foods (red meat, poultry, fishery products, eggs, dairy products) (continuous), intake of plant source foods (cereals, tuber crops and potatoes, vegetables, fruits, legumes, nuts and peanuts) (continuous), serum albumin (continuous), serum total protein (continuous), fasting blood glucose (continuous), BMI (normal, overweight, or obesity), waist circumference (continuous), systolic blood pressure (continuous), diastolic blood pressure (continuous) and heart rate (continuous).

Table S5 The associations of residual energy-adjusted dietary betaine intake with dyslipidemia stratified by age groups and sex

Variables	Residual energy-adjusted betaine intake by quartiles, *OR*s (95%*CI*)				$P_{\text{-interaction}}$
	Q1	Q2	Q3	Q4	
**High TC**					
**Age groups** [a]					0.434
6-11 years	1.00 (Ref.)	1.06 (0.79, 1.43)	0.85 (0.62, 1.17)	0.66 (0.47, 0.94)	
12-17 years	1.00 (Ref.)	0.67 (0.48, 0.91)	0.60 (0.44, 0.81)	0.54 (0.40, 0.72)	
**Sex** [b]					0.642
Male	1.00 (Ref.)	0.83 (0.58, 1.19)	0.71 (0.48, 1.03)	0.51 (0.33, 0.76)	
Female	1.00 (Ref.)	0.83 (0.64, 1.08)	0.68 (0.52, 0.89)	0.60 (0.45 ,0.78)	
**High TG**					
**Age groups** [a]					0.643
6-11 years	1.00 (Ref.)	1.17 (0.88, 1.55)	1.25 (0.94, 1.66)	1.19 (0.88, 1.60)	
12-17 years	1.00 (Ref.)	1.20 (0.93, 1.54)	1.04 (0.81, 1.32)	1.07 (0.85, 1.35)	
**Sex** [b]					0.434
Male	1.00 (Ref.)	1.22 (0.86, 1.72)	1.34 (0.96, 1.87)	1.16 (0.83, 1.61)	
Female	1.00 (Ref.)	1.15 (0.92, 1.43)	1.04 (0.83, 1.29)	1.06 (0.85, 1.32)	
**Low HDL-C**					
**Age groups** [a]					0.929
6-11 years	1.00 (Ref.)	0.79 (0.50, 1.25)	0.83 (0.52, 1.32)	0.68 (0.40, 1.14)	
12-17 years	1.00 (Ref.)	1.03 (0.78, 1.36)	1.02 (0.79, 1.33)	0.87 (0.67, 1.13)	
**Sex** [b]					0.126
Male	1.00 (Ref.)	0.89 (0.61, 1.29)	0.94 (0.66, 1.32)	0.96 (0.69, 1.33)	
Female	1.00 (Ref.)	0.99 (0.72, 1.35)	0.96 (0.71, 1.31)	0.75 (0.54, 1.04)	
**High LDL-C**					
**Age groups** [a]					0.755
6-11 years	1.00 (Ref.)	0.98 (0.64, 1.50)	0.85 (0.54, 1.32)	0.77 (0.48, 1.24)	
12-17 years	1.00 (Ref.)	0.68 (0.45, 1.04)	0.53 (0.34, 0.80)	0.62 (0.42, 0.91)	
**Sex** [b]					0.591
Male	1.00 (Ref.)	0.89 (0.55, 1.45)	0.52 (0.30, 0.89)	0.54 (0.32, 0.92)	
Female	1.00 (Ref.)	0.76 (0.53, 1.10)	0.69 (0.48, 1.00)	0.70 (0.49, 1.01)	
**High non-HDL-C**					
**Age groups** [a]					0.251
6-11 years	1.00 (Ref.)	1.00 (0.71, 1.42)	0.79 (0.55, 1.14)	0.63 (0.41, 0.95)	
12-17 years	1.00 (Ref.)	0.68 (0.48, 0.94)	0.43 (0.30, 0.61)	0.50 (0.37, 0.68)	
**Sex** [b]					0.362
Male	1.00 (Ref.)	0.92 (0.63, 1.35)	0.54 (0.35, 0.83)	0.57 (0.37, 0.86)	
Female	1.00 (Ref.)	0.71 (0.53, 0.96)	0.55 (0.41, 0.74)	0.50 (0.37, 0.68)	
**High RC**					
**Age groups** [a]					0.713
6-11 years	1.00 (Ref.)	1.00 (0.61, 1.66)	0.60 (0.34, 1.06)	0.42 (0.20, 0.85)	
12-17 years	1.00 (Ref.)	0.96 (0.61, 1.51)	0.65 (0.41, 1.03)	0.45 (0.27, 0.72)	
**Sex** [b]					0.001
Male	1.00 (Ref.)	1.62 (0.93, 2.84)	1.33 (0.74, 2.39)	0.76 (0.37, 1.49)	
Female	1.00 (Ref.)	0.71 (0.48, 1.07)	0.36 (0.23, 0.57)	0.30 (0.18, 0.49)	
**Dyslipidemia**					
**Age groups** [a]					0.628
6-11 years	1.00 (Ref.)	1.05 (0.84, 1.30)	0.99 (0.79, 1.23)	0.81 (0.64, 1.03)	
12-17 years	1.00 (Ref.)	0.96 (0.80, 1.15)	0.84 (0.71, 1.00)	0.80 (0.68, 0.94)	
**Sex** [b]					0.956
Male	1.00 (Ref.)	0.99 (0.79, 1.24)	0.88 (0.70, 1.11)	0.77 (0.62, 0.97)	
Female	1.00 (Ref.)	1.00 (0.84, 1.18)	0.87 (0.74, 1.03)	0.79 (0.67, 0.94)	

[a] The models were adjusted for sex (male or female), total energy (continuous), nationality (Han or others), residence (urban or rural), educational level of the primary caregiver (illiteracy, primary school, junior high school or high school, and above), active physical activity (less than or equal to 60 minutes per day, greater than 60 minutes per day), sleep duration (less than 8 hours per day, 8-9 hours per day,

greater than or equal to 9 hours per day), smoking or exposure to second-hand smoke (everyday, 4–6 days per week, 1–3 days per week, less than 1 day per week or never), alcohol consumption (current, former, never), menstruation or spermatorrhea (yes or no), intake of animal source foods (red meat, poultry, fishery products, eggs, dairy products) (continuous), intake of plant source foods (cereals, tuber crops and potatoes, vegetables, fruits, legumes, nuts and peanuts) (continuous), serum albumin (continuous), serum total protein (continuous), fasting blood glucose (continuous), BMI (normal, overweight, or obesity), waist circumference (continuous), systolic blood pressure (continuous), diastolic blood pressure (continuous) and heart rate (continuous).

[b] The models were adjusted for age (continuous), total energy (continuous), nationality (Han or others), residence (urban or rural), educational level of the primary caregiver (illiteracy, primary school, junior high school or high school, and above), active physical activity (less than or equal to 60 minutes per day, greater than 60 minutes per day), sleep duration (less than 8 hours per day, 8-9 hours per day, greater than or equal to 9 hours per day), smoking or exposure to second-hand smoke (everyday, 4–6 days per week, 1–3 days per week, less than 1 day per week or never), alcohol consumption (current, former, never), menstruation or spermatorrhea (yes or no), intake of animal source foods (red meat, poultry, fishery products, eggs, dairy products) (continuous), intake of plant source foods (cereals, tuber crops and potatoes, vegetables, fruits, legumes, nuts and peanuts) (continuous), serum albumin (continuous), serum total protein (continuous), fasting blood glucose (continuous), BMI (normal, overweight, or obesity), waist circumference (continuous), systolic blood pressure (continuous), diastolic blood pressure (continuous) and heart rate (continuous).

Table S6 Odds ratios (95% CIs) of residual energy-adjusted dietary betaine intake with dyslipidemia after excluding the participants with missing data of covariates: sensitivity analyses

Variables	Residual energy-adjusted betaine intake by quartiles				Increments per 1 quartile	P_{trend}
	Q1	Q2	Q3	Q4		
**High TC**						
Cases/N	197 / 2373	209 / 2372	163 / 2373	125 / 2373		
OR (95% CIs)	1.00 (Ref.)	0.84 (0.67, 1.05)	0.68 (0.54, 0.86)	0.53 (0.41, 0.68)	0.81 (0.75, 0.88)	<0.001
**High TG**						
Cases/N	219 / 2373	286 / 2372	296 / 2373	279 / 2373		
OR (95% CIs)	1.00 (Ref.)	1.22 (1.00, 1.50)	1.21 (0.99, 1.48)	1.13 (0.92, 1.38)	1.03 (0.97, 1.10)	0.354
**Low HDL-C**						
Cases/N	184 / 2373	137 / 2372	154 / 2373	134 / 2373		
OR (95% CIs)	1.00 (Ref.)	0.96 (0.74, 1.23)	0.99 (0.78, 1.27)	0.83 (0.65, 1.06)	0.95 (0.88, 1.03)	0.199
**High LDL-C**						
Cases/N	103 / 2373	92 / 2372	79 / 2373	74 / 2373		
OR (95% CIs)	1.00 (Ref.)	0.72 (0.52, 0.98)	0.61 (0.44, 0.84)	0.56 (0.40, 0.78)	0.83 (0.74, 0.92)	<0.001
**High non-HDL-C**						
Cases/N	168 / 2373	152 / 2372	108 / 2373	99 / 2373		
OR (95% CIs)	1.00 (Ref.)	0.75 (0.58, 0.97)	0.53 (0.40, 0.69)	0.49 (0.37, 0.64)	0.77 (0.71, 0.84)	<0.001
**High RC**						
Cases/N	71 / 2373	82 / 2372	49 / 2373	35 / 2373		
OR (95% CIs)	1.00 (Ref.)	0.99 (0.70, 1.42)	0.61 (0.41, 0.90)	0.45 (0.29, 0.69)	0.75 (0.66, 0.86)	<0.001
**Dyslipidemia**						
Cases/N	548 / 2373	565 / 2372	536 / 2373	470 / 2373		
OR (95% CIs)	1.00 (Ref.)	1.00 (0.86, 1.16)	0.92 (0.79, 1.06)	0.78 (0.67, 0.90)	0.92 (0.88, 0.96)	<0.001

The models were adjusted for sex (male or female), age (continuous), total energy (continuous), nationality (Han or others), residence (urban or rural), educational level of the primary caregiver (illiteracy, primary school, junior high school or high school, and above), active physical activity (less than or equal to 60 minutes per day, greater than 60 minutes per day), sleep duration (less than 8 hours per day, 8-9 hours per day, greater than or equal to 9 hours per day), smoking or exposure to second-hand smoke (everyday, 4–6 days per week, 1–3 days per week, less than 1 day per week or never), alcohol consumption (current, former, never), menstruation or spermatorrhea (yes or no), intake of animal source foods (red meat, poultry, fishery products, eggs, dairy products) (continuous), intake of plant source foods (cereals, tuber crops and potatoes, vegetables, fruits, legumes, nuts and peanuts) (continuous), serum albumin (continuous), serum total protein (continuous), fasting blood glucose (continuous), BMI (normal, overweight, or obesity), waist circumference (continuous), systolic blood pressure (continuous), diastolic blood pressure (continuous) and heart rate (continuous).

Table S7 Odds ratios (95% CIs) of residual energy-adjusted dietary betaine intake with dyslipidemia after adjusting more covariates: sensitivity analyses

Variables	Residual energy-adjusted betaine intake by quartiles				Increments per 1 quartile	P_{trend}
	Q1	Q2	Q3	Q4		
**High TC**						
Cases/N	239/2683	251/2683	198/2683	162/2683		
OR (95% CIs)	1.00 (Ref.)	0.84 (0.68, 1.03)	0.67 (0.54, 0.83)	0.56 (0.45, 0.70)	0.82 (0.77, 0.88)	<0.001
**High TG**						
Cases/N	278/2683	358/2683	349/2683	347/2683		
OR (95% CIs)	1.00 (Ref.)	1.18 (0.98, 1.42)	1.12 (0.94, 1.34)	1.11 (0.93, 1.33)	1.02 (0.97, 1.08)	0.456
**Low HDL-C**						
Cases/N	212/2683	164/2683	178/2683	159/2683		
OR (95% CIs)	1.00 (Ref.)	0.96 (0.76, 1.21)	0.98 (0.78, 1.23)	0.86 (0.68, 1.08)	0.96 (0.89, 1.03)	0.241
**High LDL-C**						
Cases/N	117/2683	117/2683	95/2683	98/2683		
OR (95% CIs)	1.00 (Ref.)	0.80 (0.60, 1.07)	0.64 (0.47, 0.86)	0.65 (0.48, 0.87)	0.86 (0.78, 0.94)	0.001
**High non-HDL-C**						
Cases/N	196/2683	187/2683	133/2683	128/2683		
OR (95% CIs)	1.00 (Ref.)	0.80 (0.62, 1.00)	0.56 (0.44, 0.71)	0.53 (0.42, 0.68)	0.80 (0.74, 0.86)	<0.001
**High RC**						
Cases/N	88/2683	99/2683	59/2683	40/2683		
OR (95% CIs)	1.00 (Ref.)	1.00 (0.72, 1.38)	0.59 (0.41, 0.84)	0.41 (0.28, 0.61)	0.73 (0.65, 0.83)	<0.001
**Dyslipidemia**						
Cases/N	664/2683	693/2683	636/2683	584/2683		
OR (95% CIs)	1.00 (Ref.)	0.99 (0.87, 1.14)	0.88 (0.77, 1.01)	0.80 (0.69, 0.91)	0.92 (0.88, 0.96)	<0.001

The models were adjusted for sex (male or female), age (continuous), and total energy (continuous), nationality (Han or others), residence (urban or rural), educational level of the primary caregiver (illiteracy, primary school, junior high school or high school, and above), active physical activity (less than or equal to 60 minutes per day, greater than 60 minutes per day), sleep duration (less than 8 hours per day, 8-9 hours per day, greater than or equal to 9 hours per day), smoking or exposure to second-hand smoke (everyday, 4–6 days per week, 1–3 days per week, less than 1 day per week or never), alcohol consumption (current, former, never), menstruation or spermatorrhea (yes or no), intake of animal source foods (red meat, poultry, fishery products, eggs, dairy products) (continuous), intake of plant source foods (cereals, tuber crops and potatoes, vegetables, fruits, legumes, nuts and peanuts) (continuous), serum albumin (continuous), serum total protein (continuous), fasting blood glucose (continuous), BMI (normal, overweight, or obesity), waist circumference (continuous), systolic blood pressure (continuous), diastolic blood pressure (continuous), heart rate (continuous), history of hypertension (yes or no), history of diabetes (yes or no), family history of hypertension (yes or no), and family history of diabetes (yes or no).

Table S8 Odds ratios (95% CIs) of energy-adjusted dietary betaine intake with dyslipidemia: sensitivity analyses

Variables	Energy-adjusted betaine intake by quartiles*				Increments per 1 quartile	P_{trend}
	Q1	Q2	Q3	Q4		
**High TC**						
Cases/N	235 / 2863	258 / 2863	204 / 2863	153 / 2863		
OR (95% CIs)	1.00 (Ref.)	0.88 (0.73, 1.08)	0.72 (0.58, 0.88)	0.52 (0.41, 0.65)	0.81 (0.75, 0.86)	<0.001
**High TG**						
Cases/N	289 / 2863	348 / 2863	336 / 2863	359 / 2863		
OR (95% CIs)	1.00 (Ref.)	1.18 (0.99, 1.40)	1.07 (0.89, 1.27)	1.10 (0.92, 1.32)	1.02 (0.96, 1.07)	0.571
**Low HDL-C**						
Cases/N	200 / 2863	176 / 2863	182 / 2863	155 / 2863		
OR (95% CIs)	1.00 (Ref.)	1.09 (0.87, 1.36)	1.05 (0.84, 1.31)	0.95 (0.75, 1.20)	0.98 (0.91, 1.06)	0.647
**High LDL-C**						
Cases/N	112 / 2863	124 / 2863	100 / 2863	91 / 2863		
OR (95% CIs)	1.00 (Ref.)	0.94 (0.72, 1.24)	0.73 (0.55, 0.97)	0.63 (0.46, 0.85)	0.85 (0.77, 0.93)	0.001
**High non-HDL-C**						
Cases/N	194 / 2863	194 / 2863	138 / 2863	118 / 2863		
OR (95% CIs)	1.00 (Ref.)	0.87 (0.70, 1.08)	0.59 (0.47, 0.75)	0.49 (0.38, 0.64)	0.78 (0.72, 0.84)	<0.001
**High RC**						
Cases/N	91 / 2863	101 / 2863	51 / 2863	43 / 2863		
OR (95% CIs)	1.00 (Ref.)	1.00 (0.74, 1.35)	0.50 (0.35, 0.71)	0.42 (0.28, 0.61)	0.72 (0.64, 0.81)	<0.001
**Dyslipidemia**						
Cases/N	658 / 2863	699 / 2863	634 / 2863	586 / 2863		
OR (95% CIs)	1.00 (Ref.)	1.05 (0.93, 1.20)	0.90 (0.79, 1.03)	0.81 (0.71, 0.93)	0.93 (0.89, 0.97)	<0.001

* Energy-adjusted betaine intake was calculated as the dietary betaine intake per day divided by the total daily energy intake.

The models were adjusted for sex (male or female), age (continuous), total energy (continuous), nationality (Han or others), residence (urban or rural), educational level of the primary caregiver (illiteracy, primary school, junior high school or high school, and above), active physical activity (less than or equal to 60 minutes per day, greater than 60 minutes per day), sleep duration (less than 8 hours per day, 8-9 hours per day, greater than or equal to 9 hours per day), smoking or exposure to second-hand smoke (everyday, 4–6 days per week, 1–3 days per week, less than 1 day per week or never), alcohol consumption (current, former, never), menstruation or spermatorrhea (yes or no), intake of animal source foods (red meat, poultry, fishery products, eggs, dairy products) (continuous), intake of plant source foods (cereals, tuber crops and potatoes, vegetables, fruits, legumes, nuts and peanuts) (continuous), serum albumin (continuous), serum total protein (continuous), fasting blood glucose (continuous), BMI (normal, overweight, or obesity), waist circumference (continuous), systolic blood pressure (continuous), diastolic blood pressure (continuous) and heart rate (continuous).

Table S9 Odds ratios (95% CIs) of residual energy-adjusted dietary betaine intake with dyslipidemia using 24-h dietary recalls method: sensitivity analyses

Variables	Residual energy-adjusted betaine intake by quartiles using the 24-h dietary recalls method				Increments per 1 quartile	P_{trend}
	Q1	Q2	Q3	Q4		
**High TC**						
Cases/N	247 / 2847	242 / 2847	225 / 2847	129 / 2848		
OR (95% CIs)	1.00 (Ref.)	0.86 (0.71, 1.04)	0.82 (0.67, 1.00)	0.50 (0.40, 0.63)	0.82 (0.77, 0.88)	<0.001
**High TG**						
Cases/N	337 / 2847	349 / 2847	313 / 2847	327 / 2848		
OR (95% CIs)	1.00 (Ref.)	1.01 (0.86, 1.20)	0.91 (0.76, 1.08)	0.95 (0.80, 1.14)	0.98 (0.92, 1.03)	0.383
**Low HDL-C**						
Cases/N	199 / 2847	171 / 2847	182 / 2847	157 / 2848		
OR (95% CIs)	1.00 (Ref.)	0.91 (0.73, 1.13)	0.94 (0.75, 1.18)	0.80 (0.63, 1.01)	0.94 (0.87, 1.01)	0.093
**High LDL-C**						
Cases/N	122 / 2847	113 / 2847	120 / 2847	68 / 2848		
OR (95% CIs)	1.00 (Ref.)	0.78 (0.60, 1.03)	0.86 (0.65, 1.12)	0.50 (0.36, 0.68)	0.83 (0.76, 0.92)	<0.001
**High non-HDL-C**						
Cases/N	195 / 2847	178 / 2847	167 / 2847	99 / 2848		
OR (95% CIs)	1.00 (Ref.)	0.80 (0.64, 1.00)	0.75 (0.60, 0.94)	0.46 (0.36, 0.60)	0.80 (0.74, 0.86)	<0.001
**High RC**						
Cases/N	94 / 2847	66 / 2847	70 / 2847	54 / 2848		
OR (95% CIs)	1.00 (Ref.)	0.69 (0.50, 0.95)	0.74 (0.53, 1.03)	0.58 (0.40, 0.82)	0.85 (0.76, 0.95)	0.005
**Dyslipidemia**						
Cases/N	701 / 2847	686 / 2847	635 / 2847	538 / 2848		
OR (95% CIs)	1.00 (Ref.)	0.99 (0.87, 1.14)	0.88 (0.77, 1.01)	0.80 (0.69, 0.91)	0.92 (0.88, 0.96)	<0.001

The models were adjusted for sex (male or female), age (continuous), and total energy (continuous), nationality (Han or others), residence (urban or rural), educational level of the primary caregiver (illiteracy, primary school, junior high school or high school, and above), active physical activity (less than or equal to 60 minutes per day, greater than 60 minutes per day), sleep duration (less than 8 hours per day, 8-9 hours per day, greater than or equal to 9 hours per day), smoking or exposure to second-hand smoke (everyday, 4–6 days per week, 1–3 days per week, less than 1 day per week or never), alcohol consumption (current, former, never), menstruation or spermatorrhea (yes or no), intake of animal source foods (red meat, poultry, fishery products, eggs, dairy products) (continuous), intake of plant source foods (cereals, tuber crops and potatoes, vegetables, fruits, legumes, nuts and peanuts) (continuous), serum albumin (continuous), serum total protein (continuous), fasting blood glucose (continuous), BMI (normal, overweight, or obesity), waist circumference (continuous), systolic blood pressure (continuous), diastolic blood pressure (continuous) and heart rate (continuous).
